# Screening for *in-vivo* regional contractile defaults to predict the delayed Doxorubicin Cardiotoxicity in Juvenile Rat

**DOI:** 10.7150/thno.47407

**Published:** 2020-07-09

**Authors:** Nourdine Chakouri, Charlotte Farah, Stefan Matecki, Pascal Amedro, Marie Vincenti, Laure Saumet, Laurence Vergely, Nicolas Sirvent, Alain Lacampagne, Olivier Cazorla

**Affiliations:** 1 PHYMEDEXP, INSERM, CNRS, Université de Montpellier, CHRU Montpellier, Montpellier, France.; 2 FATH-IREC, Université Catholique de Louvain, Brussel, Belgium.; 3 Pediatric and Congenital Cardiology Department, M3C Regional Reference CHD Centre, University Hospital, Montpellier, France.; 4 Service d'onco-hématologie pédiatrique, CHRU Montpellier, Montpellier, France.; 5 Unité de Pharmacie Clinique Oncologique, CHRU Montpellier, Montpellier, France.

**Keywords:** Anthracyclines, cardiotoxicity, regional speckle tracking echocardiography, heart failure, myofilament

## Abstract

Anthracyclines are key chemotherapeutic agents used in various adult and pediatric cancers, however, their clinical use is limited due to possible congestive heart failure (HF) caused by acute and irreversible cardiotoxicity. Currently, there is no method to predict the future development of the HF in these patients. In order to identify early biomarkers to predict anthracycline cardiotoxicity in long-term survivors of childhood cancer, this longitudinal study aimed to analyze early and late *in-vivo* regional myocardial anthracycline-induced cardiotoxicity, related to *in-vitro* cardiac myocytes dysfunction, in a juvenile rat model.

**Methods:** Young male Wistar rats (4 weeks-old) were treated with different cumulative doses of doxorubicin (7.5, 10 or 12.5 mg/kg) or NaCl (0.9%) once a week for 6 weeks by intravenous injection. Cardiac function was evaluated *in-vivo* by conventional (left ventricular ejection fraction, LVEF) and regional two-dimensional (2D) speckle tracking echocardiography over the 4 months after the last injection. The animals were assigned to preserved (pEF) or reduced EF (rEF) groups at the end of the protocol and were compared to controls.

**Results:** We observed a preferential contractile dysfunction of the base of the heart, further altered in the posterior segment, even in pEF group. The first regional alterations appeared 1 month after chemotherapy. Functional investigation of cardiomyocytes isolated from the LV base 1 month after doxorubicin treatment showed that early *in-vivo* contractile alterations were associated with both decreased myofilament Ca^2+^ sensitivity and length-dependent activation. Changes in post-translational modifications (phosphorylation; S-glutathionylation) and protein degradation of the cardiac myosin binding protein-C may contribute to these alterations.

**Conclusion:** Our data suggest that screening of the contractile defaults of the base of the heart by regional 2D strain echocardiography is useful to detect subclinical myocardial dysfunction prior to the development of delayed anthracycline-induced cardiomyopathy in pediatric onco-cardiology.

## Introduction

Anthracyclines (including doxorubicin) are key chemotherapeutic agents used in various adult and pediatric cancers. Their clinical use is however limited due to possible congestive heart failure (HF) 1-3 caused by acute and irreversible cardiotoxicity 4. The cumulative doses of anthracyclines and the young age of patients receiving these medications mainly impact the occurrence of the delayed irreversible cardiotoxicity 1,4,5. This cardiotoxicity is more complex than a dose-dependence effect, as some patients with low doses (below 240mg/m²) have demonstrated subclinical cardiac abnormalities 6,7. The heterogeneity of patient sensitivity - comorbidities, genetic and concomitant environmental risks factors - may predispose for HF after chemotherapy treatment 8. Advances in therapeutic strategies for childhood cancer have favored a growing number of long-term survivors that are compromised by side-effects. Among them, congestive HF remains the most significant medical complication 4,9. As such, guidelines in cancerology and cardiology recognize the need for greater awareness of cardiotoxicity progression 9,10.

Currently, pediatricians are calling for a non-invasive early detection strategy to predict the development and risk of this delayed HF 11. Circulating biomarkers such as cardiac troponin I (cTnI) and N-terminal B-type natriuretic peptide have shown promising results in adult patients but have not been validated in pediatric clinical trials 12. Consequently, the routine practice in clinic to detect anthracycline-related cardiotoxicity is regular assessment of left ventricular ejection fraction (LVEF) measured by conventional echocardiography 13. Nevertheless, it may detect the rare instances of acute cardiotoxicity in pediatrics but fail to predict cases of late onset myocardial dysfunction 14. Recently, LV global longitudinal 2D strain echocardiography (*i.e.* speckle tracking echocardiography (STE)) has emerged as a tool for detecting anthracycline-related myocardial dysfunction before LVEF depression occurs in adult patients 15-17. STE is now recommended by the European and American Societies of Echocardiography as a routine component of clinical follow-up in patients at risk for cardiotoxicity 18. Even if the STE analysis allows the evaluation of the global, but also regional, cardiac strains, the existing studies using STE only focused on global LV strain and did not routinely evaluated regional strain indexes as potential predictors of chemotherapy-induced cardiotoxicity. In pediatric patients, investigation of STE as a prognostic biomarker was only performed in small cohorts and did not provide regional STE analysis 19. Furthermore, clinical studies should include prospective cohorts of pediatric and adult patients over larger period of time, as cardiomyopathy usually occurs after several decades. The animal models use may favor investigation of STE as a biomarker over a shorter duration. However, to date, most pre-clinical studies were performed in adult animals, reporting acute or sub-acute cardiotoxicity (*i.e.* less than 12 weeks) obtained by single bolus and/or intra-peritoneal anthracycline injections, but did not examine delayed cardiac damages.

Multiple mechanistic pathways have been proposed to explain cardiac side-effects 20. Anthracycline-induced cardiotoxicity was proposed to be mediated by the topoisomerase 2β poisoning, which alters mitochondrial function and subsequently triggers apoptosis and massive reactive oxygen species (ROS) production in cardiomyocytes 20,21. Other cardiotoxic effects proposed include necrosis, inflammation, myofilament protein dysfunction, extracellular matrix remodeling, and intracellular Ca^2+^ dysregulation 7,22,23. These anthracycline-mediated cell injuries lead to contractile impairment, irreversible myocardial damage and fibrosis. More recently, carbonylation and degradation of the sarcomeric protein cardiac myosin binding protein C (cMyBPC) have been identified as a cause of anthracycline cardiotoxicity 24, but the functional impact remains unclear.

Our study aimed to determine if regional STE analysis could be used as a prognostic biomarker of delayed anthracycline-induced cardiomyopathy. We investigated the evolution of global and regional cardiac function through several months after a 5-week period of doxorubicin treatment in juvenile rats, at both *in-vivo* and *in-vitro* contractile machinery level.

## Methods

For additional details in methods, please see Extended Methods in Supplementary Materials.

### Animal studies and experimental design

**Figure 1:**
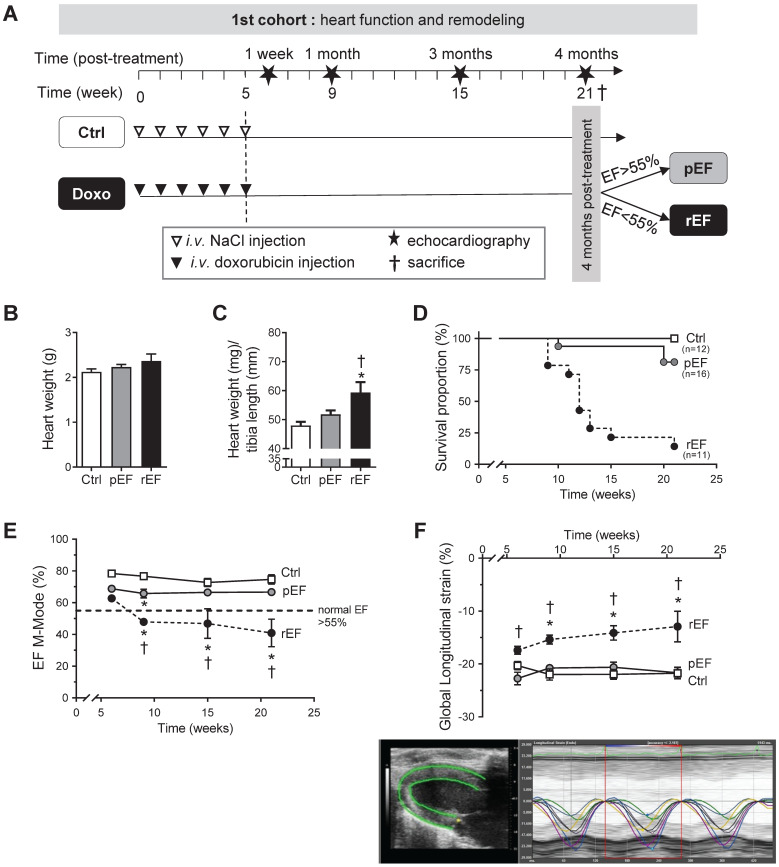
** Effect of doxorubicin on cardiac function and mortality.** (**A**) Schematic representation of the *in-vivo* animal model of delayed doxorubicin cardiotoxicity. Animals were injected with saline solution (Ctrl, white triangle) or doxorubicin (Doxo, black triangle), weekly at different cumulative doses for five consecutive weeks. Echocardiography (star) was performed 1 week, 1 month, 3 months and 4 months after the last injection. At the end of the protocol, animals were separated according to their LV ejection fraction (EF): preserved EF above 55% (pEF group, n=16) or reduced LVEF below 55% (rEF group, n=11) and were compared with Ctrl animals (n=12). Animals were sacrificed after the last echocardiography for *in-vitro* explorations (black cross). (**B**) Heart weight in animals 4 months after doxorubicin treatment or at ethical limit-point sacrifice compared to Ctrl. (**C**) Heart weight to tibia length ratios in animals 4 months after doxorubicin treatment. (**D**) Survival proportion curves of Ctrl (open square), pEF (grey circle), and rEF (black circle) animals (log-rank test for trend, P=0.001). (**E**) Evolution of LVEF in Ctrl, pEF and rEF animals along the protocol. (**F**) LV global longitudinal strain measured by STE echocardiography. Bottom panel: representative image of LV longitudinal strain with the six LV segments recorded, allowing an *a posteriori* manual calculation of our model of mean LV global longitudinal strain values. *, p<0.05 *vs* Ctrl; ^†^, p<0.05 *vs* pEF; ANOVA followed by Tukey *post-hoc* tests.

Young male Wistar rats (4 weeks-old, n=82; weight=96.4±1.9 g, Janvier Laboratories, Le-Genest-Saint-Isle, France) were housed on a 12-hour light-dark cycle with free access to water and food. All investigations complied with European Directive 2010/63/EU and were approved by our institutional ethics committee on animal research (CEEA-00950.01). Doxorubicin treatment was based on clinical practice, literature 25, and preliminary experiments to adjust doses adapted to delayed cardiotoxicity in juvenile rats. We aimed to obtain, over a short period of time, the different clinical presentations of anthracycline-induced cardiotoxicity in rats, which would be observed in human over several decades. To wit one group of animals developing a delayed cardiomyopathy with reduced ejection fraction (rEF) within the post-treatment period, and one group maintaining a preserved ejection fraction (pEF) under the same experimental conditions, independently of the administrated dose (Figure 1A). Animals received doxorubicin weekly by intra-venous injection (bolus, tail vein) during 6 consecutive weeks, in order to reach 3 cumulative doses: 7.5 mg/kg, 10mg/kg, and 12.5 mg/kg. Control (Ctrl) rats were treated with saline solution (0.9% NaCl), following the same procedure. Echocardiography follow-up was performed at 1 week, 1 month, 3 months and 4 months post-chemotherapy. At the end of the protocol, LVEF was determined to include animals into the pEF group or the rEF group. Considering the animals growth during the experiments, we used LVEF cut-offs used in clinical practice to define myocardial dysfunction (below 55%) 11. In a second set of experiments, the protocol ended 1 month after the last injection. Unless ethical endpoints were reached, animals were sacrificed under pentobarbital sedation at 1 or 4 months after the last anthracycline injection (Figure 1A and Figure 3A).

### High-resolution echocardiography

Transthoracic echocardiography using a high-resolution ultrasound system (Vevo 2100; VisualSonics, Toronto, Canada) assessed cardiac function and morphology in anesthetized rats (1-3% isoflurane, 100% oxygen). We used a 40-MHz linear array transducer (MS550, VisualSonics) in young animals (< 200g) and subsequently a 21-MHz linear array transducer (MS250, VisualSonics). During the echocardiography, body temperature was maintained at 37°C and ECG monitoring ensured physiological heart rate (> 350 bpm).

#### Standard echocardiography

LV parasternal long axis 2D view in M-mode was performed at the level of papillary muscle to assess LV wall thicknesses and internal diameters, LV fractional shortening (FS), ejection fraction (EF) and relative wall thickness index (RWT). LV diastolic function was estimated by assessment of transmitral inflow waves by pulse-wave Doppler measured in the apical four-chamber view, completed by tissue Doppler imaging of the mitral annulus. Pulse-wave Doppler of the ascending aorta was recorded to measure the aortic velocity time integral (AoVTI). The measurements were analyzed from three cardiac cycles using the VevoLab software (VisualSonics), following the current guidelines 26 (Table S1).

#### Speckle tracking echocardiography

**Figure 2:**
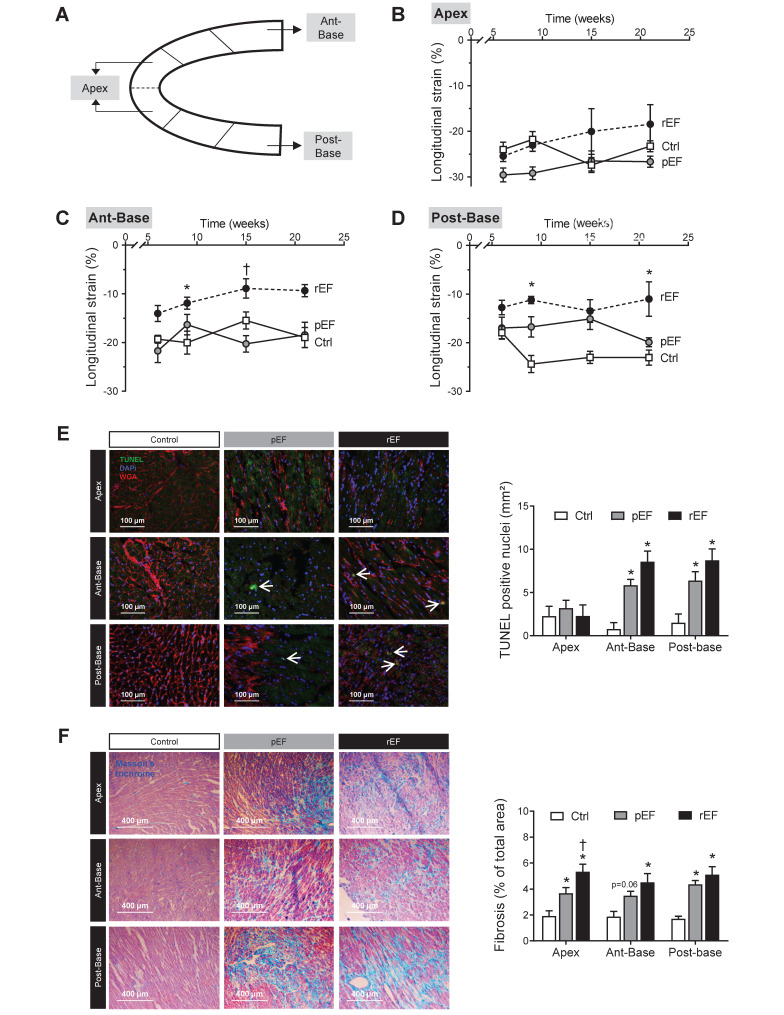
** Regional cardiac function and remodeling 4 months after doxorubicin treatment.** (**A**) Representative image of LV longitudinal 2D strain and selected segments analyzed: (**B**) LV apex, (**C**) LV anterior-base, and (**D**) LV posterior-base measured in pEF (grey circle, n=9) and rEF (black circle, n=11) animals 1 week, 1 month, 3 months and 4 months after doxorubicin treatment compared with Ctrl animals (open square, n=9). (**E**) Representative histological images (cross section) of hearts after TUNEL staining from LV apex (*top*), anterior-base (*middle*) and posterior-base (*bottom*) in Ctrl, pEF and rEF groups, 4 months after chemotherapy treatment. Wheat germ agglutinin (WGA) stains myocyte membranes (red), DAPI stains nuclei (blue), apoptotic nuclei are shown in green. Bar graph shows quantification of apoptosis by positively-stained nuclei (number/mm^2^) 4 months after Doxo treatment in Ctrl (n=3), pEF (n=5) and rEF (n=4) animals. (**F**) Representative histological images (cross section) of hearts after Masson trichrome staining from LV apex (*top*), anterior-base (*middle*) and posterior-base (*bottom*) in Ctrl, pEF and rEF groups. Bar graph shows quantification of LV fibrosis (percentile of total area) 4 months after doxorubicin treatment in Ctrl (n=3), pEF (n=5) and rEF (n=4) animals. *, p<0.05 *vs* Ctrl; ^†^, p<0.05 *vs* pEF; ANOVA followed by Tukey *post-hoc* tests.

For STE analysis, parasternal long axis views were recorded in B-mode by adjusting the scanning range to achieve optimal visualization of the whole LV myocardium with the highest frame rate. Analyses were performed by a blinded single trained investigator. The endocardial and epicardial borders were traced manually. The VevoStrain software automatically delimited six equally-spaced myocardial segments and calculated a 2D strain curve for each (Figure 1G). The quality of tracking was visually validated. Due to ribs shadowing, the LV anterior and posterior middle segments had irregular tracking and were excluded from the analysis. Thus, we focused on three LV myocardial segments: the basal anterior (ant-base), the basal posterior (post-base), and the apex obtained as the average of the anterior and posterior apex regions (Figure 2A). Each value is averaged from 2-3 cine loops, and each cine loop from 2-3 cardiac cycles. LV global strain is the average of these 3 regions.

### Histological analysis

After animal sedation hearts were immediately removed, rinsed and frozen in isopentane. Transverse sections (5 µm thickness) were performed at the cardiac base and apex levels using a cryostat (Microm, HM560). Cardiac fibrosis was quantified using Masson's Trichrome staining that colored cell cytoplasm in red, cell nuclei in purple, and collagen in blue. Fibrosis was calculated by dividing the total area of blue staining (collagen) by the total area of the LV using ImageJ software and the threshold color plugin. Apoptosis was assessed by *in situ* terminal deoxynucleotidyl transferase (TdT)-mediated digoxigenin-conjugated deoxyuridine triphosphate (dUTP) nick end-labeling (TUNEL), (DeadEnd™ Fluorometric TUNEL kit, Promega, Madison, USA), completed with wheat germ agglutinin (WGA 1:250; Vector Labs) and 4′,6-diamidino-2-phenylindole (DAPI 1:1,000; Sigma-Aldrich) staining. The number of TUNEL-positive cardiomyocytes was defined as the number of labeled positive cardiomyocytes per section divided by the total area (mm²). Fibrosis and apoptosis were quantified from 3 different sections per region (n=3-5 hearts per group).

### Force measurement in permeabilized cardiomyocytes

Isometric force was measured in single cardiomyocytes permeabilized with Triton X-100 as previously described 27,28. Small pieces of tissues were dissected from the apex, the posterior-base and the anterior-base and mechanically dissociated. Force was measured at different Ca^2+^ concentration ([Ca^2+^]) expressed as pCa (= -log[Ca^2+^]), at 1.9 then 2.3 µm sarcomere length (SL) (n= 5-6 hearts per group). Force was normalized to the cross-sectional area measured by imaging (IonOptix system, Hilton, USA). The relationship between Ca^2+^-activated force and pCa was fitted to a Hill equation. The Ca^2+^-activating solutions contained: 12 mmol/L phosphocreatine, 30 mmol/L imidazole, 1 mmol/L free Mg^2+^, 10 mmol/L EGTA, 3.3 mmol/L Na_2_ATP, 0.3 mmol/L dithiothreitol, and protease inhibitors to prevent degradation (0.5 mmol/L PMSF, 0.04 mmol/L leupeptin, and 0.01 mmol/L E64 [trans-epoxysuccinyl-1-leucine-guanidobutylamide]), with a pH of 7.1 adjusted with acetic acid.

### Western blot analysis

LV frozen tissue was powdered in tissue grinder on dry-ice and solubilized in a non-reducing Laemmli buffer 27. cMyBPC and cTnI expressions and phosphorylations, and S-glutathionylation of cMyBPC were studied as previously described (n=4-5 hearts per group) 29,30. Primary antibodies are detailed in Table S5. After incubation with fluorescent secondary antibodies, bands were quantified with the Odyssey system (LI-COR Biosciences, Lincoln, USA).

### Statistical analysis

Statistics were performed using GraphPad Prism software (GraphPad Software, La-Jolla, USA). Data were presented using mean ± SEM values. Data were subjected to Student* t*-test or one-way or two-way ANOVA test. When significant interactions were found, a Tukey *post hoc* test was applied with p< 0.05.

## Results

### Doxorubicin induces different levels of global cardiac alteration in rats

Four months after the end of chemotherapy protocol, LVEF was measured and animals were assigned to the pEF or the rEF groups (Figure 1A). No major cardiac remodeling was observed after doxorubicin treatment (Figure 1B, Table S1). The heart weight/tibia length ratio was however increased in the rEF group, suggesting a relative cardiac hypertrophy mainly explained by animal growth alterations (Figure 1C; see tibia length in Table S1), but also related with a slight cardiomyocyte hypertrophy (Supplemental Figure S1). In the pEF group, we observed a trend for a LV concentric hypertrophic remodeling (Figure 1 B-C and see RWT in Table S1), which was associated with higher cardiomyocytes size (Supplemental Figure S1). The mortality rate was higher in the rEF group (Figure 1D). The conventional echocardiography follow-up revealed that in the rEF group the LVEF started to decrease 1 month after the last doxorubicin injection and further declined during the four months to 41±3% (Figure 1E). Myocardial relaxation was altered only in the rEF group, related to a higher cardiac stiffness (reduced e' and increased E/e'; Table S1). Global LV longitudinal 2D strain decreased only in the rEF group (Figure 1F; top panel) and was lower compared with pEF groups at all-time points, even 1 week after the end of doxorubicin treatment.

### Doxorubicin induces regional alterations of the LV

Next, we analyzed the evolution of cardiac regional longitudinal 2D strain (Figure 2A). No significant difference was observed between doxorubicin-treated animals and controls for the apex longitudinal 2D strain, with a relatively stable value around -25% throughout the protocol (Figure 2B). The basal anterior 2D strain of the rEF group was reduced compared to both pEF and control groups (Figure 2C), following a similar reduction pattern as the global LV longitudinal 2D strain (Figure 1F). One month after chemotherapy treatment, the basal posterior longitudinal 2D strain of rEF and pEF group were gradually decreased (-11% and -17%, respectively) compared with control animals (-25%) and remained reduced until the end of the protocol (Figure 2D). The basal posterior segment thus clearly showed regional defects of cardiac contractility which could not be detected by conventional echocardiography or STE global analysis in the pEF group.

At the end of the protocol, to link functional defaults with cellular alterations, we assessed apoptosis by TUNEL staining in the different regions (Figure 2E). Apoptosis was absent in the apex while it was increased in both regions of the base in pEF animals compared with control animals (Figure 2E) and was further amplified in rEF animals. Doxorubicin-treated animals had more myocardial fibrosis compared to control animals (Figure 2F). In pEF animals, the level of fibrosis increased from the apex to the anterior-base and further in the posterior-base (4.6%), while the higher fibrosis levels were observed in all cardiac regions of rEF animals (about 5% in the three regions). These results indicate that doxorubicin induced deleterious regional alterations in all animals, even in animals with preserved EF and normal global LV strain (summarized in Table S2).

### Doxorubicin induces regional alterations of myocyte mechanics at early stages of HF

**Figure 3:**
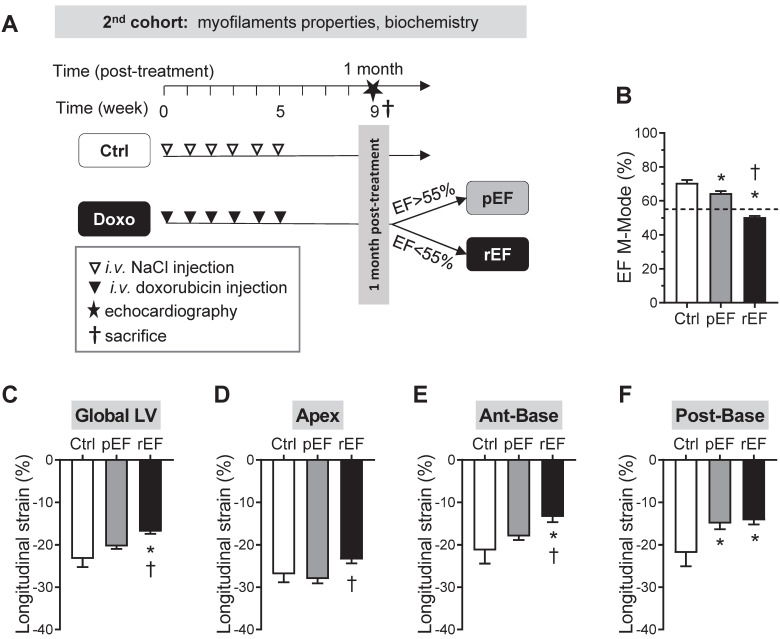
** Regional cardiac function at early stages after doxorubicin treatment.** Schematic representation of the experimental procedure to detect early modifications of doxorubicin cardiotoxicity (1 month after treatment), following similar protocol than for the first cohort of animals. (**B-F**) Evaluation of LVEF (B), Global LV (C), LV apex (D), LV anterior-base (E), and LV posterior-base (F) longitudinal strain measured in Ctrl animals (black column, n=6), pEF (grey column, n=18) and rEF (open column, n=12) animals 1 month after they received doxorubicin. *, p<0.05 *vs* Ctrl; ^†^, p<0.05 *vs* pEF; ANOVA followed by Tukey *post-hoc* tests.

**Figure 4:**
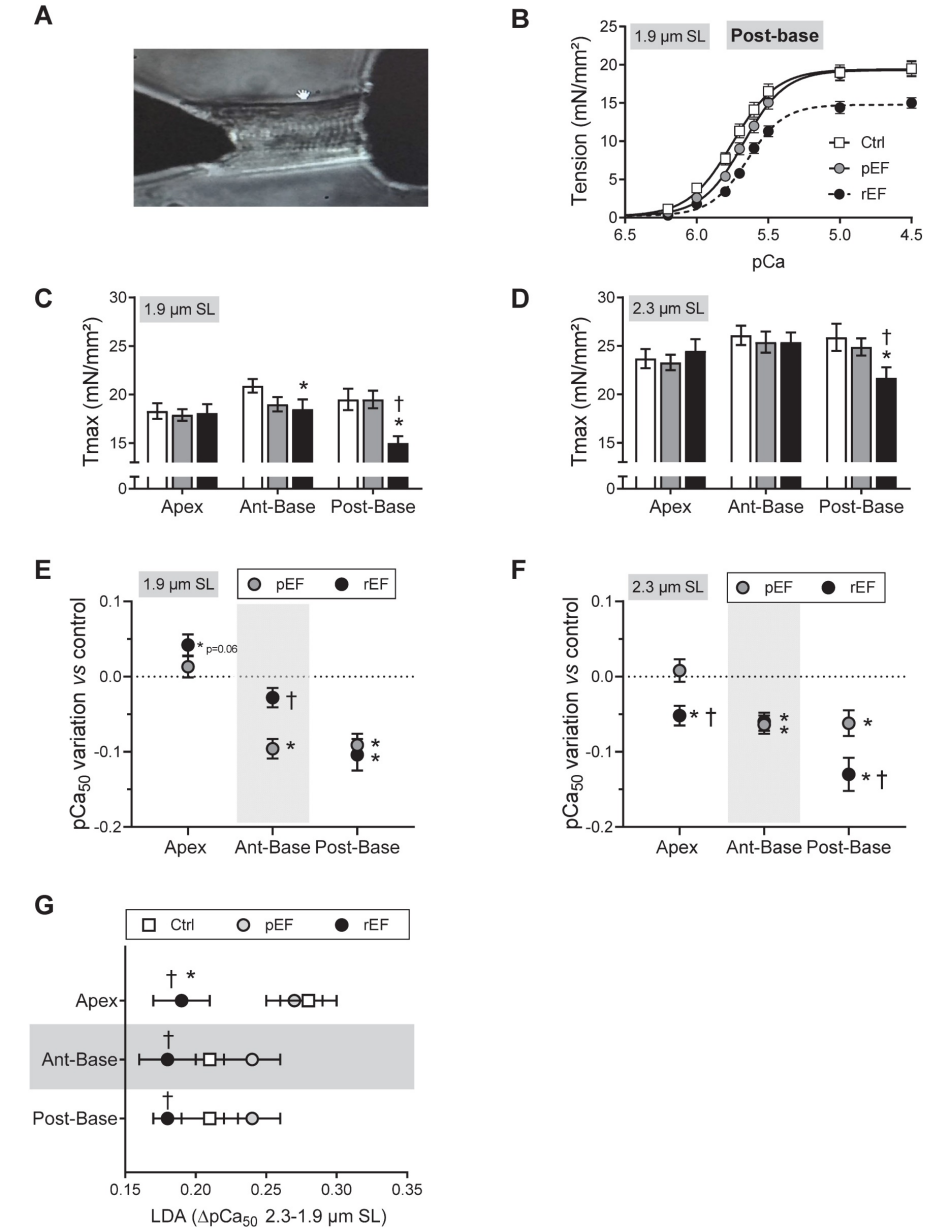
** Regional effect of doxorubicin on myofilaments contractile properties at early stages of cardiotoxicity.** (**A**) Representative image of permeabilized cardiomyocyte attached to a force transducer. (**B**) Evaluation of tension - Ca^2+^ relationship at short sarcomere length (SL) in permeabilized cardiomyocytes isolated from LV posterior-base in Ctrl, pEF and rEF animals. (**C-D**) Evaluation of maximal tension measured in Ctrl (open bar), pEF (grey bar) and rEF (black bar) permeabilized cardiomyocytes, at 1.9 µm (C) and 2.3 µm (D) SL. (**E-F**) Variation of the pCa_50_ (pCa for half maximal activation) obtained from the normalized tension-pCa curves, of pEF (gray circle) and rEF (black circle) cardiomyocytes, relative to the Ctrl values at 1.9 µm (E) and 2.3 µm (F), in permeabilized cardiomyocytes. (**G**) Evaluation of the stretch-induced Ca^2+^ sensitization (Δ pCa_50_) index of the length-dependent activation (LDA) calculated from the difference in pCa_50_ obtained at 2.3 and 1.9 µm SL, in each experimental condition. n=18-28 cells/5-6 hearts per condition. *, p<0.05 *vs* Ctrl; ^†^, p<0.05 *vs* pEF; ANOVA followed by Tukey *post-hoc* tests.

We investigated the cellular and molecular mechanisms responsible for the early significant cardiac contractile changes that occur since 1 month after chemotherapy (Figure 1E). As previously, animals were assigned to the pEF or rEF group according to their LVEF measurements at the end of the short protocol (Figure 3A-B; Table S3). Importantly, animals exhibited similar changes to animals from the prior protocol, regarding both global LV longitudinal 2D strain (Figure 3C) and all cardiac segments (Figure 3D-F). These results confirm the reliability and reproducibility of the changes in regional 2D strains analysis. The specific properties of the contractile machinery were studied in permeabilized myocytes isolated from the 3 various LV regions (Figure 4). The relationship between the Ca^2+^-activated tension and the amount of Ca^2+^ was established at short (1.9µm) and long (2.3µm) SL in cardiomyocytes to cover the physiological working range (Figure 4 and Table S4). The maximal active tension (Tmax) generation decreased only in rEF animals. In this group, the posterior-base was the most affected at both short (-23%) and long (-16%) SL when compared with control myocytes (Figure 4C-D; Table S4). The anterior-base of rEF animals seemed less affected with a slight but significant (-11%; p=0.05) decrease of Tmax only at short SL (Figure 4C; Table S4). Myofilament Ca^2+^ sensitivity was measured by assessing the pCa required to develop half of the maximal tension (pCa_50_). The effects of doxorubicin treatment were expressed relative to control values to facilitate interpretation (Figure 4E-F and Figure S2). Myofilaments of pEF myocytes were less sensitive to Ca^2+^ in both segments of the LV base at short (Figure 4E) and long SL (Figure 4F) (Table S4). In rEF animals, myofilament Ca^2+^ sensitivity was reduced only in the posterior-base at short SL (Figure 4E; Table S4) but was reduced in all regions at long SL compared with controls (Figure 4F). The largest changes of pCa_50_ were observed in the posterior-base. The difference of pCa_50_ between long and short SL (ΔpCa_50_, Figure 4G), termed length**-**dependent activation (LDA), reflects a physiological regulatory mechanism of the heart to adapt to stretching by increasing myofilament Ca^2+^ sensitivity. Interestingly, LDA of rEF myofilaments was lower in all cardiac segments when compared with pEF myocytes (Figure 4G; Table S4). Altogether, these results indicate that doxorubicin induces regional modifications in contractile machinery, even in animals with normal LVEF. We observed a gradient of preferential regional dysfunction from the apex to the base, with larger alterations in the posterior-base.

**Figure 5:**
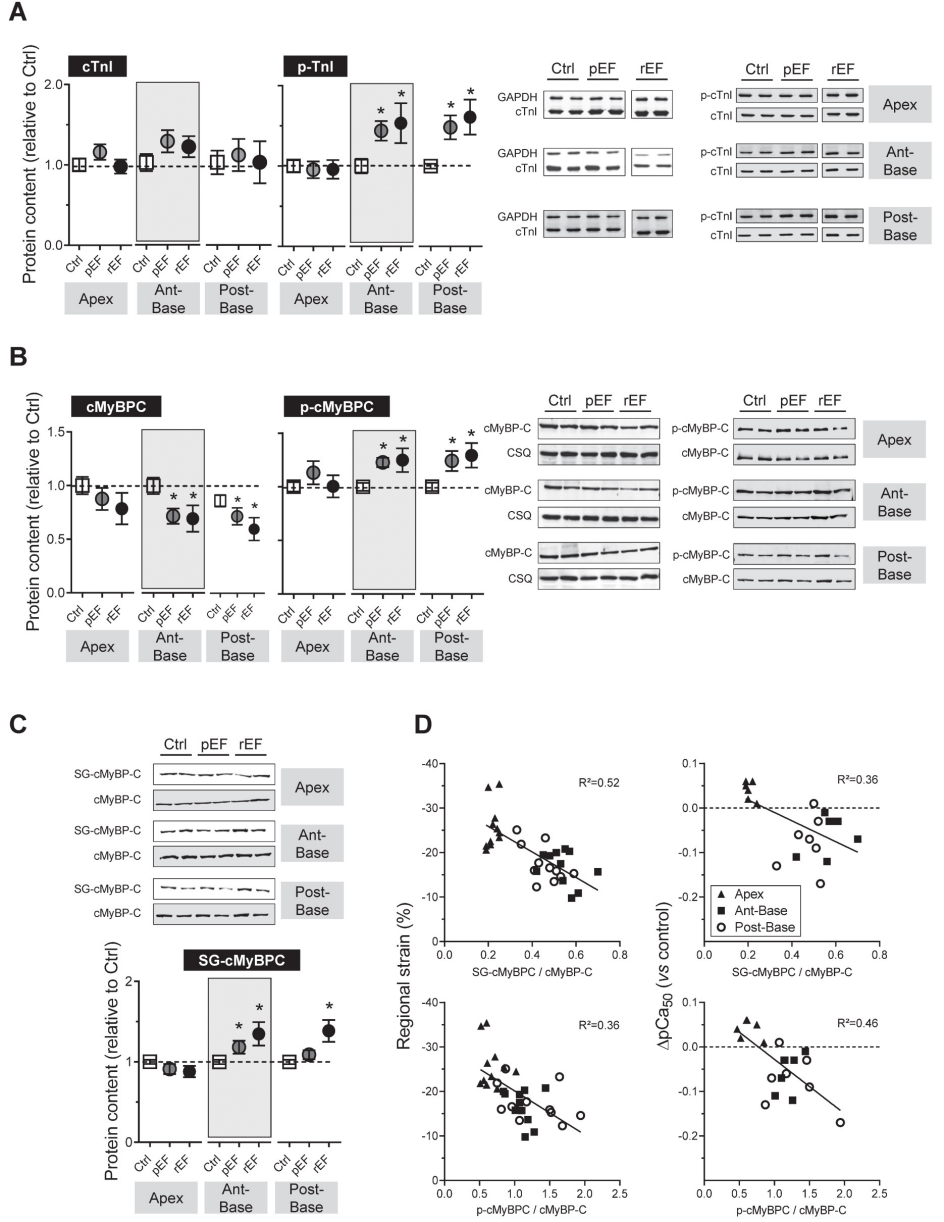
** Regional effect of doxorubicin on sarcomeric proteins post-translational modification at 1 month after doxorubicin treatment.** (**A-C**) Regulatory contractile proteins modifications were evaluated by Western blot analysis. Evaluation of cTnI content and cTnI phosphorylation (p-cTnI) at Ser23/24 (A), cMyBP-C content and cMyBP-C phosphorylation (p-cMyBP-C) at Ser282 (B), and cMyBP-C S-glutathionylation (C) in myocardial samples from LV apex (left), LV anterior-base (middle) and LV posterior-base (right) in Ctrl, pEF and rEF groups. Level cTnI was expressed relative to Glyceraldehyd-3-phosphat dehydrogenase (GAPDH) and level of cMyBP-C was expressed relative to calsequestrin. Levels of phosphorylated cMyBP-C (p-cMyBP-C) and TnI (p-TnI) were normalized to the total content of cMyBP-C and TnI respectively. Level of cMyBP-C S-glutathionylation (SG-cMyBPC) was normalized to total cMyBP-C. *Note that in panel A, images illustrating the p-cTnI/cTnI blots is a composite image obtained from the same blot*. n=4 hearts per condition. (**D**) Left panels: Correlations between the *in-vivo* regional strain of each segment and the degree of post-translational modifications of cMyBP-C glutathionylation (top panel) and cMyBP-C phosphorylation (bottom panel). Right panels: Correlations between the *in-vitro* variation of myofilaments Ca^2+^ sensitivity of treated groups relative to control and the degree of post-translational modifications of cMyBP-C glutathionylation (top panel) and cMyBP-C phosphorylation (bottom panel) *, p<0.05 *vs* Ctrl; ^†^, p<0.05 *vs* pEF; ANOVA followed by Tukey *post-hoc* tests.

To gain insight in the signaling mechanisms underlying the differences in contractile machinery properties between pEF and rEF animals, protein degradation and post-translational modifications of cTnI and cMyBP-C were investigated (Figure 5). Since doxorubicin had no effect on the sarcomeric regulatory proteins isolated from apex cardiomyocytes, we discuss below only the results obtained on the LV basal segments. One month after chemotherapy, we did not observe any protein degradation of cTnI (Figure 5A). However, the expression of cMyBP-C was reduced in both segments of the base of rEF animals. A similar trend was observed in pEF animals (Figure 5B). The phosphorylation levels of cTnI and cMyBP-C at PKA sites, known to be activated by ROS-dependent mechanisms in pathological condition 27,31, increased significantly in both segments of the base of pEF and rEF animals, to a similar extent in both groups (Figure 5 A-B). Finally, the level of S-glutathionylated cMyBP-C, a reversible redox-sensitive post-translational modification 32, increased in the whole LV base in rEF animals (~35-40%) compared to controls, and increased only in the anterior-base in pEF animals (~20% increase) (Figure 5C).

Moreover, we found relevant correlations between the *in-vivo* regional strain of each segment and the degree of post-translational modifications of cMyBP-C (Figure 5D, left panels). Similar correlations were also found between the *in-vitro* variation of myofilaments Ca^2+^ sensitivity of treated groups relative to control (Figure 5D, right panels). These correlations revealed that decreases of strain *in-vivo* and myofilaments Ca^2+^ sensitivity *in-vitro* of a myocardial segment are related to an increase of glutathionylation and phosphorylation of cMyBP-C.

## Discussion

Detecting cardiac side-effects of childhood cancer therapies before the occurrence of irreversible LV dysfunction is essential. In adults, 2D strain echocardiography is a promising diagnosis tool 15-17,33-36 but needs large-scale studies for validation in young patients. Moreover, clinical practice and previous studies 34-36 analyzed only the LV global 2D strain to predict chemotherapy-induced cardiotoxicity, and its link with underlying sarcomere dysfunction is poorly known. The originality of our work was to take full advantage of STE analysis by correlating the *in-vivo* contractility of the different cardiac segments to cellular regional contraction.

### Doxorubicin induces heterogeneous alterations of cardiac function

In accordance with clinical observations 1,6, we observed a dose-dependent cardiotoxicity of doxorubicin and also heterogeneous sensitivity to treatment with an HF occurrence of 15%, 48%, and 75% (LVEF< 55%) in juvenile rats receiving 7.5, 10 and 12mg/kg of doxorubicin, respectively. We demonstrated that normal LVEF and/or global LV strain values can hide regional dysfunctions and we highlighted that LV posterior-base myocardium was preferentially altered after chemotherapy, even in animals with normal LVEF. Validation of LV longitudinal strain as a sensitive marker to detect early subclinical LV alterations in childhood cancer survivors remains to be demonstrated due to the lack of large-scale study. But in the recent years some small cohort studies have shown altered LV global longitudinal strain in adolescent and/or young adults with normal EF few months 37, few years 38,39 and more than a decade 40,41 after chemotherapy. These studies corroborate our observations in our rodent model. However, in these studies, 2D STE parameters were evaluated only at one-time point and did not investigate the LV longitudinal strain evolution over years after anthracyclines treatment. Our work offers an original approach to the regional LV strains kinetic, which allowed us to detect early segmental alterations in the cardiac base, even before the decline of global LV strain. Moreover, the reproducibility of the early changes of regional 2D strains analysis between our 2 cohorts of animals support the reliability of this non-invasive screening tool for the detection of delayed anthracyclines cardiotoxicity. Abnormal LV posterior-base function has been reported previously during chemotherapy in adolescent patients follow-up 42. In this study, regional contractility decreased in the apical, middle, and basal segment 4 months after the beginning of chemotherapy. Interestingly, all segments recovered at 8 months except the basal segment, which could be responsible for global LV function impairments. Alteration of the LV longitudinal strain of the basal segment, at the septum, but not of the apical region, was also observed in adolescent survivors of childhood cancer with normal conventional echocardiography 39. Similarly, hypokinesia of the LV basal area was observed in other cardiomyopathies such as in Duchenne muscular dystrophy 43 and in Miyoshi myopathy patients with preserved LV function 44, and was associated with more extensive fibrosis than elsewhere. Amount of myocardial fibrosis is an independent prognostic of greater risk of adverse cardiac disease outcome 45. This is consistent with our observations of higher apoptosis in basal segments, light global fibrosis, and more pronounced fibrosis in the posterior-base. Although we cannot explain why the LV base is preferentially altered after chemotherapy, previous studies have proposed that during ventricular filling, higher mechanical stress is exerted at the heart base 46. A characteristic progressive reduction of the LV strain from the base to the apex was observed in restrictive cardiomyopathy associated with increased myocardial stiffness 47. This asymmetric constraint could be responsible for the preferential decrease of LV longitudinal strain and not the radial and circumferential strains in hypertrophic cardiomyopathy 47. In our study, we also evaluated radial and circumferential strains, but it did not display additional interest on longitudinal strain. To the opposite, the study of Cheung *et al*. showed in anthracyclines-treated survivors of childhood cancers, some impairment of LV twisting and untwisting motion mostly due to defaults in the apical rotation 48. Similar defaults were found in the study of Yu *et al.* that also found basal rotation decrease in their young adult patients without alteration of longitudinal strain 49. This discrepancy with our results could be explained by myocardial layer-specific structural differences between human and rodent hearts. Moreover, despite the reliability and reproducibility of our regional STE measurements, we cannot exclude some specific technical limitations of speckle tracking sensitivity for cardiac rodents investigations (high HR; ribs shadowing; very high frequency ultrasound probes) of myocardial sublayers strains or specific cardiac segments (exclusion of middle segment in this study).

### Doxorubicin induces cellular contractile alterations at early stages of HF

The molecular mechanisms of chemotherapy-induced cardiotoxicity remain poorly understood. Among the multiple mechanisms proposed, all agree for a fundamental role of ROS generation in this cardiotoxicity 7,21-23. ROS modifications are known to affect contractile properties of cardiomyocytes, notably by inducing redox-dependent direct or indirect post-translational modifications of sarcomeric proteins 27,50. In this study, we observed oxidative stress in the whole heart after chemotherapy, especially in rEF hearts, as indicated by the increase of MDA levels (Figure S3), a marker of lipid peroxidation. During conditions of prolonged oxidative stress, protein carbonylation, an irreversible post-translational modification, can alter sarcomeric proteins activity or promote protein degradation 24,51,52. Total protein carbonylation profile (high and low molecular weight) was increased in the whole LV base of the rEF animals (Figure S3 E-F) while in the apex only proteins at high molecular weight (>250 kDa) were affected (Figure S3 E). A recent study showed specific carbonylation and degradation of cMyBP-C two weeks after a single chemotherapy treatment, thus associating modifications of cMyBP-C with cardiac dysfunction 24. This is consistent with our findings showing degradation of cMyBP-C in the base one month after chemotherapy (Figure 5B). Interestingly, we previously showed that the cMyBP-C deficiency using a transgenic mouse model decreases length-dependent activation of myofilaments 53. Thus, the alteration of myofilament LDA by doxorubicin could be partly due to lower cMyBP-C content and could contribute to early heart contractile dysfunction.

The specific involvement of cMyBP-C after chemotherapy is reinforced by our study showing an increase in S-glutathionylation of the protein, a reversible ROS-dependent modification. Moreover, we found important correlations between the *in-vivo* (strain) and *in-vitro* (myofilament Ca^2+^ sensitivity) regional contractile properties and the levels of cMyBP-C phosphorylation and S-glutathionylation (Figure 5D). These results suggest that *in-vitro* and *in-vivo* contractile properties are impaired at early stages through a redox-dependent mechanism. Together, these results support a link between doxorubicin-induced ROS production and altered cardiac contractility mediated by cMyBP-C post-translational modifications in cardiomyocytes.

Several limitations in our study warrant comments. First, the animals used in the present study do not have cancer tumors. Thus, they do not have all of the modifications inherent to cancer pathophysiology. Nevertheless, the knowledge/understanding of the isolated mechanisms of action of doxorubicin at an early stage of the disease is of great importance to prevent additional health problems in patients with cancer. Secondly, here we have tested only the effect of doxorubicin on cardiotoxicity. However, other classes of anti-cancer drugs like HER2-inhibitor are commonly used alongside anthracyclines as a combination therapy, due to enhanced anti-cancer efficacy. But, often this combination unfortunately leads to an increased risk of cardiotoxicity 54, which remains to be tested**.** Finally, in our model of delayed anthracyclines-cardiotoxicity, it would be interesting to test whether this inevitable cardiotoxicity could be reduced by new therapeutic processing. Several strategies have been tested to reduce side effects of doxorubicin by either changing the delivery mode and solubility *via* encapsulation of the drug 55, or improving the targeting to tumors and enhancing anticancer efficacy of doxorubicin 56, or combining treatments with cardioprotective molecules against inflammation or oxidative stress 57.

In conclusion, we showed that doxorubicin exposure induces a regionalized cardiac dysfunction initiated early at the LV posterior base, even in animals with normal EF, which may extend to the whole base of the heart. This could further lead to heart failure. Moreover, this study establishes a causal relationship between doxorubicin-induced cardiomyocytes dysfunction and heart dysfunction, suggesting that early cellular alteration is causal to HF evolution and not the consequence of pathological cardiac remodeling. We propose that regional STE could be a reliable non-invasive tool for the detection of early subclinical myocardial dysfunction before the development of delayed anthracycline-cardiomyopathy, essential for therapeutic intervention and adjustment in pediatric cancer patients.

## Supplementary Material

Supplementary methods, figures and tables.

## Abbreviations

Ant-base: anterior base
Ca^2+^: calcium
EF: ejection fraction
GAPDH: glyceraldehyde-3-phosphate dehydrogenase
HF: heart failure
LV: left ventricle
LVEF: left ventricular ejection fraction
MDA: malondialdehyde
cMyBP-C: cardiac myosin binding protein-C
pEF: preserved ejection fraction
PKA: protein kinase A
Post-base: posterior base
rEF: reduced ejection fraction
ROS: reactive oxygen species
SL: sarcomere length
STE: speckle tracking echocardiography
cTnI: cardiac Troponin I
