# Supplementary material for: Screening for in-vivo regional contractile defaults to predict the delayed Doxorubicin Cardiotoxicity in Juvenile Rat
Source: Theranostics. 2020 Jul 9;10(18):8130–42. doi: 10.7150/thno.47407 (PMC7381739; doi:10.7150/thno.47407)
Supplement: Supplementary file 1 — Supplementary methods, figures and tables. [file thnov10p8130s1.pdf]

Supplementary Materials

Screening for in-vivo regional contractile defaults to predict the delayed doxorubicin cardiotoxicity in juvenile rat

Nourdine Chakouri [1, †], Charlotte Farah [1,2 †], Stefan Matecki [1], Pascal Amedro [1,3], Marie Vincenti [1,3], Laure Saumet [4], Laurence Vergely [5], Nicolas Sirvent [3], Alain Lacampagne [1], Olivier Cazorla [1,]

[1] PHYMEDEXP, INSERM, CNRS, Université de Montpellier, CHRU Montpellier, Montpellier, France ;

[2] FATH-IREC, Université Catholique de Louvain, Brussel, Belgium ;

[3] Pediatric and Congenital Cardiology Department, M3C Regional Reference CHD Centre, University Hospital, Montpellier, France ;

[4] Service d'onco-hématologie pédiatrique, CHRU Montpellier, Montpellier, France ;

[5] Unité de Pharmacie Clinique Oncologique, CHRU Montpellier, Montpellier, France.

[†] Both authors contributed equally to this work.

Table of contents

Expanded Methods……………………………………………………………………………. 2

Supplementary Table S1-S5…………………………………………………………... 6

Supplementary Figure S1-S3……….……………….………………………………………. 11

Full non edited blots……….………………………………………………………………… 15

Reference…..........…………………………………………………………………….... 18

Expanded Methods

Animal studies and experimental design. Young male Wistar rats (4 weeks-old, n=82; weight=96.4±1.9g; Janvier Laboratories, Le Genest-Saint-Isle, France) were housed on a 12-hour light-dark cycle and had free access to water and food. All investigations complied with European regulations (European Parliament Directive 2010/63/EU) and were approved by our institutional ethics committee on animal research ("Comité d'éthique pour l'expérimentation animale Languedoc-Roussillon", number CEEA-00950.01). Dose selection was based on clinical practice, literature, and preliminary experiments to adjust doxorubicin doses adapted to delayed cardiotoxicity in juvenile rats. Indeed, 5mg/kg Doxo did not show *in-vivo* cardiotoxicity effects. The dose of 15mg/kg induced a very high and rapid mortality rate in young animals, as previously observed by Moulin *et al* in adult rats [1], which suggests that this dose is adapted to study acute but not delayed cardiotoxicity. The goal of the study was to obtain similar phenotype variations after Doxo treatment in rats compared to human studies, where one group of animals develops a delayed cardiomyopathy with reduced ejection fraction (rEF) within the 4-month period after treatment, and one group preserves ejection fraction (pEF) under the same experimental conditions, independently of the administrated dose (Fig. 1A). A total of 66 animals were treated weekly with Doxo (provided by our institutional pharmacy department) by intra-venous injection (bolus, tail vein) during 6 consecutive weeks, in order to reach 3 cumulative doses: 7.5mg/kg, 10mg/kg, and 12.5mg/kg. Control rats were treated with saline solution (0.9% NaCl), following the same procedure. Echocardiography follow-up was performed 1 week, 1 month, 3 months and 4 months post-chemotherapy, respectively. At the end of the protocol (*i.e.* 4 months after the last injection), animals were separated into 2 groups according to the determined LVEF: the group with preserved ejection fraction group (pEF) and the group with reduced ejection fraction group (rEF). Considering the growth of the animals during the experiments, we used a cut-off value of LVEF below 55% to define myocardial dysfunction, as in clinical practice[2]. In a second set of experiments, the protocol ended 1 month after the last injection. Unless ethical endpoints were reached, animals were sacrificed under pentobarbital sedation at 1 or 4 months after the last anthracycline injection (Fig. 1A and Fig. 3A).

High-resolution echocardiography. Cardiac function and morphology were assessed in anesthetized rats (1-3% isoflurane, 100% oxygen) by transthoracic echocardiography using a high-resolution ultrasound system (Vevo 2100; VisualSonics, Toronto, Canada) equipped with a 40-MHz linear array transducer (MS550, Vevo2100, VisualSonics) for the 2 first evaluations

(animal weight < 200g) and a 21-MHz linear array transducer (MS250, Vevo 2100, VisualSonics) subsequently to the end of the protocol. Animals were placed on a heated table in a supine position. Body temperature was maintained at 37°C and ECG was recorded all along the echocardiographic procedure with limb electrodes to ensure physiological heart rate (> 350 bpm).

Standard echocardiography. LV parasternal long axis 2D view in M-mode was performed at the level of papillary muscle to assess LV wall thicknesses and internal diameters, allowing the calculation of the fractional shortening (FS) and ejection fraction (EF) by the Teicholz method. The relative wall thickness index (RWT) was calculated as RWT= (IVSd+PWTd)/LVIDd (with IVS: inter-ventricular septum; PWT: posterior wall thickness; LVID: LV internal diameter, d = in diastole). Parasternal long axis views at papillary muscle level in B-mode were also recorded by adjusting the scanning range to achieve optimal visualization of all the LV myocardium with the highest frame rate for STE analysis. LV diastolic function was estimated by the transmitral pulse-wave Doppler measured in the apical four-chamber view by placing the sample at the tip of the mitral valves. Velocity peaks of early (E) and late atrial contraction (A) mitral inflow waves were measured and the E/A ratio was calculated. Tissue Doppler imaging of the mitral annulus was performed to assess e' (early diastolic myocardial relaxation velocity) and a' (late diastolic myocardial relaxation velocity) waves. The ratio E/e' was calculated as an index of LV filling pressure. Finally, pulse-wave Doppler of the ascending aorta was recorded to measure the aortic velocity time integral (AoVTI) (Table S1). All measurements were quantified using the VevoLab software (VisualSonics), and the results were averaged over three cardiac cycles, following the current guidelines [3].

Speckle tracking echocardiography. Global and regional left ventricular dynamics were assessed by speckle tracking analysis (e.g. 2D strain) from B-mode cine loops acquired during the echocardiographic sessions. Parasternal long axis views were used for the evaluation of LV longitudinal 2D strain. 2D strain analyses were performed by a single trained investigator, blinded to group assignment and treatment allocation. The investigator manually traced the endocardial and epicardial borders, the VevoStrain software (VisualSonics) automatically delimited six equally-spaced tissue tracking myocardial segments, and 2D strain curves were traced for each segment. The quality of tracking was visually validated. Considering the high tracking variability of the LV anterior and posterior middle segments due to ribs shadowing in parasternal long axis view, these LV regions were excluded from the analysis. Thus, we focused on three LV myocardial segments: basal-anterior (ant-base), the basal posterior (post-base), and

the apex obtained as the average of the anterior and posterior apex regions (Fig. 2A). LV global strain was then manually calculated as the mean of these 3 regions. Each value was obtained by the mean of 2 or 3 cine loops, and for each cine loop, 2 to 3 cardiac cycles were examined.

Western blot analysis. Myocardial protein expression was studied as previously described [4]. Briefly, LV tissue was quickly frozen in liquid nitrogen until biochemical analysis. Frozen tissue was powdered in tissue grinder on dry-ice and solubilized in a non-reducing Laemmli buffer (3% SDS, 50mmol/L Tris HCl pH 6.8, 8mol/L urea, 2mol/L thiourea, 1mmol/L EGTA, 1mmol/L EDTA, 10mmol/L Benzamidine, 0.2mmol/L Na_3VO_4, 50mmol/L NaF, 20mmol/L β-glycerophosphate). Proteins were separated using SDS-PAGE electrophoresis and transferred onto nitrocellulose membranes (GE Healthcare). Then, membranes were blocked with the Odyssey Blocking Buffer in PBS (LI-COR Biosciences) at room temperature for 45min and incubated overnight with primary antibodies (Table S4). Bands were revealed and quantified with the Odyssey system (LI-COR Biosciences, Lincoln, Nebraska) after incubation with fluorescent secondary antibodies.

S-glutathionylation of cardiac myosin-binding protein-C. S-glutathionylation of cMyBP-C was measured using non-reducing SDS-PAGE as previously described [5,6]. Briefly, samples were solubilized in non-reducing Laemmli buffer (3% SDS, 50mmol/L Tris HCl pH 6.8, 8mol/L urea, 2mol/L thiourea, 1mmol/L EGTA, 1mmol/L EDTA, 10mmol/L Benzamidine, 0.2mmol/L Na_3VO_4, 50mmol/L NaF, 20mmol/L β-glycerophosphate) with 25mmol/L Nethylmaleimide (NEM) during 45min at room temperature. Proteins were resolved on 10% acrylamide SDS-PAGE and transferred onto nitrocellulose membranes as described above (GE Healthcare). After blocking, membranes were incubated overnight with primary antibodies (anti-GSH, anti-cMyBP-C3, and anti-CSQ). For each sample, the glutathionylated band (140kDa) was normalized to the level of total cMyBP-C on the same gel (n=4 hearts per group).

Malondialdehyde assay (Figure S1). As an index of myocardial lipid peroxidation, malondialdehyde (MDA) levels were measured in cardiac tissue using the Lipid Peroxidation (MDA) Assay Kit (ab118970, Abcam, Cambridge, Mass, United States), according to the manufacturer's instructions. Briefly, small LV pieces were homogenized in lysis buffer and directly centrifuged at 15,000 g at 4°C for 15 min. The resulting supernatants were used to measure MDA content by spectrofluorimetry (n=3-5 hearts per condition).

Protein carbonylation (Figure S2). Total myocardial protein carbonylation was measured using the Oxyblot Protein Oxidation Detection kit (Millipore Corporation, Massachusetts) as previously described [4]. Briefly, carbonyl groups in samples containing 15 µg proteins were derivatized by reaction with 2,4-dinitrophenylhydrazine (DNP) for 15 min. Dinitrophenyl-derivatized proteins were separated on 10% SDS-PAGE gels and transferred to nitrocellulose membranes (GE Healthcare). Next, membranes were incubated overnight with primary antibodies, a rabbit anti-Dinitrophenyl and a mouse anti-GAPDH. Bands were revealed with the Odyssey system (LI-COR Biosciences, Lincoln, Nebraska) after incubation with fluorescent secondary antibodies (45 min at room temperature, 1: 30,000 dilution, LI-COR Biosciences). Carbonylated bands were normalized to the level of GAPDH (n= 4 hearts per condition).

Table S1: Effects of doxorubicin on animals' growth and cardiac morphology and function assessed by echocardiography 4 months after the end of treatment or early endpoint.

	Ctrl	pEF	rEF
	Animals morphologic parameters		
**n**	12	16	14
**BW (g)**	614 ± 24	470 ± 23 *	359 ± 25 *†
**HW (g)**	2.12 ± 0.07	2.23 ± 0,06	2.36 ± 0,16
**TL (mm)**	44.2 ± 0.3	43.1 ± 0,4	39.7 ± 0,6 *†
	Echocardiographic parameters		
**n**	12	16	11
**HR (bpm)**	374 ± 9	362 ± 9	352 ± 13
**IVSd (mm)**	1.8 ± 0.1	2.0 ± 0.1	1.7 ± 0.1†
**IVSs (mm)**	3.2 ± 0.1	3.2 ± 0.1	2.4 ± 0.1 *†
**LVIDd (mm)**	7.8 ± 0.2	8.1 ± 0.1	8.6 ± 0.3
**LVIDs (mm)**	4.3 ± 0.2	5.0 ± 0.1	6.9 ± 0.4 *†
**PWTd (mm)**	2.0 ± 0.1	2.1 ± 0.1	1.9 ± 0.1†
**PWTs (mm)**	3.3 ± 0.1	3.3 ± 0.04	2.5 ± 0.1*†
**RWT**	0.44 ± 0.03	0.51 ± 0.02	0.42 ± 0.02†
**FS (%)**	44 ± 2	38 ± 1 *	21 ± 2 *†
**EF (%)**	74 ± 2	66 ± 1 *	41 ± 3 *†
**MV E (mm/s)**	897± 44	964 ± 36	853 ± 43
**MV A (mm/s)**	577 ± 45	614 ± 66	445 ± 118 *†
**E/A**	1.63 ± 0.11	1.62 ± 0.17	1.65 ± 0.45
**e' (mm/s)**	77 ± 2	67 ± 3	39 ± 5 *†
**a' (mm/s)**	49 ± 5	43 ± 6	33 ± 9
**E/e'**	11.7 ± 0.6	14.7 ± 0.8	27.3 ± 5.1*†
**Ao VTI (mm)**	62 ± 2	59 ± 1	49 ± 3 *†

Results are expressed as the mean ± SEM. BW: body weight; HW: heart weight; TL: tibial length; HR: heart rate; IVSd: Interventricular septum thickness in diastole; IVSs: Interventricular septum thickness in systole; LVIDd: left ventricular internal diameter in diastole; LVIDs: left ventricular internal diameter in systole; LVPWd: left ventricular posterior wall thickness in diastole; LVPWs: left ventricular posterior wall thickness in systole; RWT: relative wall thickness (calculated as RWT= ((IVSd + PWTd)/LVIDd)); FS: fractional shortening; EF: ejection fraction; MV E: velocity peak of early mitral inflow wave; MV A: velocity peak of late atrial contraction mitral inflow waves; e': velocity peak of early diastolic myocardial relaxation velocity at mitral annulus; a': velocity peak of late diastolic myocardial relaxation velocity at mitral annulus; Ao VTI: aortic valve velocity time integral; (*=p<0.05 *vs* ctrl; †=p<0.05 *vs* pEF).

<u>Table S2:</u> Overview of left ventricular regional alterations profile observed 4 months after doxorubicin treatment.

		Longitudinal Strain		Fibrosis		Apoptosis		Cell section	
Apex	**Ctrl**	-23.3 ± 1.2		1.9 ± 0.4		2.3 ± 1.1		396 ± 34	
	**pEF**	-26.7 ± 1.2	=	3.7 ± 0.4 *	↗	3.2 ± 0.9	=	588 ± 13 *	↗
	**rEF**	-18.4 ± 4.3	=	5.3 ± 0.6 *†	↗	2.3 ± 1.3	=	546 ± 36	↗
Ant-Base	**Ctrl**	-19.0 ± 2.1		1.9 ± 0.4		0.8 ± 0.8		527 ± 42	
	**pEF**	-18.4 ± 2.6	=	3.5 ± 0.3	=	5.8 ± 0.7 *	↗	731 ± 57 *	↗
	**rEF**	-9.3 ± 1.3	↘	4.5 ± 0.7 *	↗	8.6 ± 1.2 *	↗	541 ± 26 T	=
Post-Base	**Ctrl**	-23.0 ± 1.5		1.7 ± 0.2		1.5 ± 1.0		527 ± 34	
	**pEF**	-19.9 ± 0.9	↘	4.4 ± 0.3 *	↗	6.4 ± 1.0 *	↗	878 ± 56 *	↗
	**rEF**	-11.0 ± 3.5 *	↘	5.1 ± 0.6 *	↗	8.7 ± 1.3 *	↗	734 ± 82 *	↗

Longitudinal strain obtained from regional left ventricular speckle tracking echocardiography. Apoptosis and fibrosis were analyzed by TUNEL staining and Masson trichrome staining, respectively, 4 months after chemotherapy treatment. Cardiomyocyte cross section (in μm^2) was determined using histological images of hearts after wheat germ agglutinin (WGA) staining in at least 100 cells/heart (n = 3-5 animals/ region) (*p<0.05 vs Ctrl; †=p<0.05 vs pEF).

Table S3: Transthoracic echocardiography standard parameters measured before sacrifice at 1 month after the end of the treatment.

	Ctrl	pEF	rEF
**n**	6	18	12
**HR (bpm)**	353 ± 18	389 ± 7	382 ± 9
**IVSd (mm)**	1.7 ± 0.04	1.7 ± 0.04	1.8 ± 0.1
**IVSs (mm)**	3.0 ± 0.1	2.9 ± 0.04	2.6 ± 0.1 *†
**LVIDd (mm)**	8.4 ± 0.1	8.1 ± 0.1	8.1 ± 0.1
**LVIDs (mm)**	4.9 ± 0.2	5.1 ± 0.1	5.9 ± 0.1 *†
**PWTd (mm)**	2.0 ± 0.1	1.9 ± 0.04	2.0 ± 0.1
**PWTs (mm)**	3.0 ± 0.1	2.9 ± 0.1	2.7 ± 0.1
**RWT**	0.43 ± 0.02	0.45 ± 0.01	0.48 ± 0.02
**FS (%)**	42 ± 1	37 ± 1 *	27 ± 1 *†
**EF (%)**	71 ± 2	65 ± 1 *	51 ± 1 *†
**MV E (mm/s)**	965 ± 44	992 ± 29	944 ± 43
**MV A (mm/s)**	812 ± 47	661 ± 76	631 ± 154
**E/A**	1.20 ± 0.04	1.49 ± 0.15	1.65 ± 0.42
**e' (mm/s)**	70 ± 11	68 ± 4	51 ± 4 †
**a' (mm/s)**	62 ± 2	61 ± 13	47 ± 9
**E/e'**	15.8 ± 2.6	15.7 ± 1.2	19.6 ± 1.7
**Ao VTI (mm)**	82 ± 3	63 ± 2 *	53 ± 3 *†

Results are expressed as the mean ± SEM. HR: heart rate; IVSd: Interventricular septum thickness in diastole; IVSs: Interventricular septum thickness in systole; LVIDd: left ventricular internal diameter in diastole; LVIDs: left ventricular internal diameter in systole; LVPWd: left ventricular posterior wall thickness in diastole; LVPWs: left ventricular posterior wall thickness in systole; RWT: relative wall thickness ((calculated as RWT= ((IVSd + PWTd)/LVIDd)); FS: fractional shortening; EF: ejection fraction; MV E: velocity peak of early mitral inflow wave; MV A: velocity peak of late atrial contraction mitral inflow waves; e': velocity peak of early diastolic myocardial relaxation velocity at mitral annulus; a': velocity peak of late diastolic myocardial relaxation velocity at mitral annulus; Ao VTI: aortic valve velocity time integral; (*p<0.05 vs ctrl; †=p<0.05 vs pEF).

Table S4: Regional effect of doxorubicin mechanical properties at early stage.

	n	Sarcomere length. 1.9 µm				Sarcomere length. 2.3 µm				
		T_{pass}	T_{max}	pCa_{50}	n_H	T_{pass}	T_{max}	pCa_{50}	n_H	ΔpCa_{50}
**Apex**										
Ctrl	24	0.8 ± 0.1	16.1 ± 0.7	5.67 ± 0.02	3.1 ± 0.1	5.5 ± 0.5	20.9 ± 0.9	5.95 ± 0.02	2.7 ± 0.1	0.28 ± 0.02
pEF	28	0.7 ± 0.1	15.7 ± 0.5	5.69 ± 0.01	2.6 ± 0.1[a]	5.1 ± 0.5	20.5 ± 0.7	5.96 ± 0.01	2.4 ± 0.1[a]	0.27 ± 0.02
rEF	20	0.7 ± 0.1	15.9 ± 0.8	5.72 ± 0.01	3.4 ± 0.1[a.b]	5.0 ± 0.7	21.5 ± 1.1	5.90 ± 0.01[a.b]	2.7 ± 0.1[b]	0.19 ± 0.02[a.b]
**Ant-Base**										
Ctrl	24	1.2 ± 0.2	18.4 ± 0.6	5.74 ± 0.02	3.1 ± 0.2	7.6 ± 1.1	22.9 ± 0.9	5.95 ± 0.01	2.8 ± 0.1	0.21 ± 0.01
pEF	28	0.6 ± 0.1[a]	16.4 ± 0.9	5.64 ± 0.01[a]	3.0 ± 0.1	5.7 ± 0.6	21.8 ± 1.0	5.88 ± 0.01[a]	2.4 ± 0.1[a]	0.24 ± 0.02
rEF	18	0.8 ± 0.3	16.3 ± 0.8[a]	5.71 ± 0.01[b]	3.3 ± 0.2	5.5 ± 0.6	22.3 ± 1.0	5.89 ± 0.01[a]	2.8 ± 0.1[b]	0.18 ± 0.02[b]
**Post-Base**										
Ctrl	23	1.0 ± 0.2	17.2 ± 0.9	5.76 ± 0.02	3.1 ± 0.2	6.7 ± 0.7	22.1 ± 1.3	5.97 ± 0.02	2.5 ± 0.1	0.21 ± 0.02
pEF	28	0.7 ± 0.2	16.4 ± 0.8	5.67 ± 0.01[a]	3.1 ± 0.1	6.6 ± 0.8	21.2 ± 0.8	5.90 ± 0.02[a]	2.6 ± 0.1	0.24 ± 0.02
rEF	20	0.3 ± 0.1[a.b]	13.2 ± 0.6[a.b]	5.65 ± 0.02[a]	3.6 ± 0.2	4.8 ± 0.6	19.1 ± 1.0[a.b]	5.84 ± 0.02[a.b]	3.0 ± 0.2	0.18 ± 0.01[b]

Mechanical properties of LV posterior-base, anterior-base and apex permeabilized cells isolated from Ctrl, pEF and rEF hearts. Myofilament Ca^{2+} sensitivity represented by pCa for half-maximal activation (pCa_{50}) and n_H (Hill coefficient) at 1.9 and 2.3 µm sarcomere length were calculated by fitting the force–pCa relation. Passive (T_{pass}) and maximal active (T_{max}) tensions in mN/mm^2 were measured at pCa 9 and 4.5 respectively, at 1.9 µm and 2.3 µm sarcomere length and normalized to the cross-sectional area measured by imaging (IonOptix system). Values are expressed as mean ± SEM (n = number of cells; ^{a}=p<0.05 vs ctrl; ^{b}=p<0.05 vs pEF)

<u>Table S5:</u> Antibody details

Antibody	Concentration	Source	Manufacturer
TnI	1 :5,000	Mouse	Hytest (4T21)
TnI(p-$S^{23/24}$)	1 :10,000	Rabbit	Abcam (ab58545)
cMyBP-C3	1 :1,000	Mouse	Santa Cruz Biotech.(sc-137181)
cMyBP-C3 (p-S^{282})	1 :1,000	Rabbit	Enzo LS (ALX-215-057)
GSH	1 :1,000	Mouse	Virogen (101-A)
Calsequestrin	1 :5,000	Rabbit	Thermo Fisher (PA1-913)
GAPDH	1 :60,000	Mouse	Abcam (ab8245)
DNP	1 :300	Rabbit	Merk Millipore (S7150)

Supplemental Figure 1

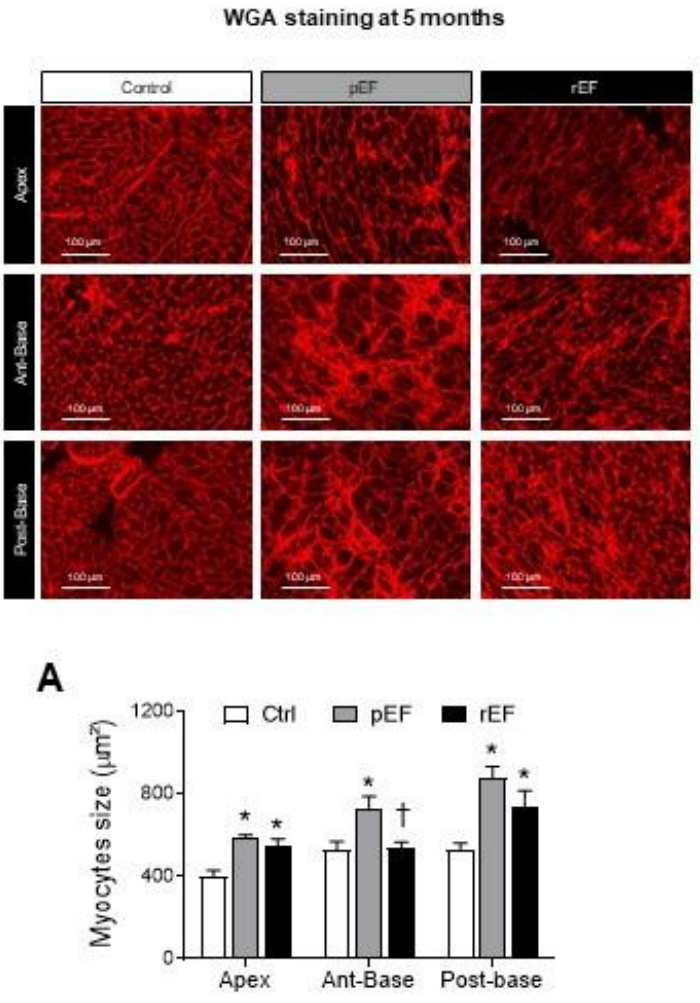

<u>Figure S1.</u> Regional effect of doxorubicin on cardiac remodeling at 4 months after the end of treatment. Images show representative histological cross section of hearts after WGA staining from LV apex (*top*), anterior-base (*middle*) and posterior-base (*bottom*) in Ctrl, pEF and rEF groups, 4 months after chemotherapy treatment. (**A**) Quantification of cardiomyocyte size (in μm^2) was determined in at least 100 cells/heart (n = 3-5 animals/ region). *, $p<0.05$ *vs* Ctrl; †, $p<0.05$ *vs* pEF; ANOVA followed by Tukey *post-hoc* tests.

Supplemental Figure 2

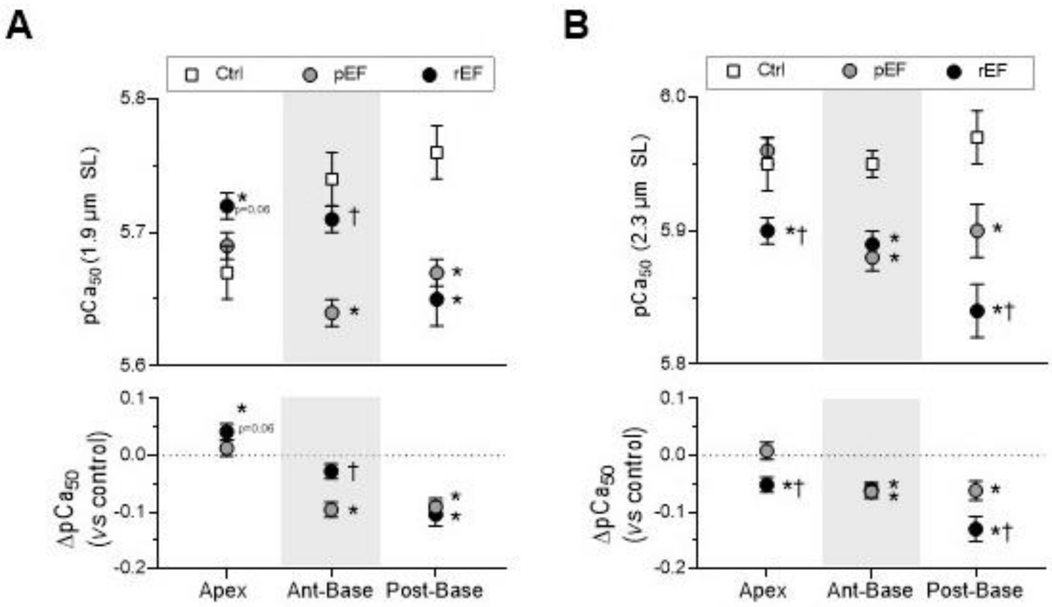

<u>**Figure S2.**</u> **Regional effect of doxorubicin on myofilaments contractile calcium sensitivity at early stages of cardiotoxicity.** Cardiomyocytes were isolated from LV apex, LV anterior-base and LV posterior-base, permeabilized and attached to a force transducer. **Top panels**: Myofilament Ca^{2+} sensitivity indexed by pCa_{50} (pCa for half maximal activation) obtained from the normalized tension-pCa curves, in Ctrl (open square), pEF (gray circle) and rEF (black circle) groups cardiomyocytes, at 1.9 µm (**A**) and 2.3 µm (**B**) SL. **Bottom panels**: Variation of the pCa_{50} of pEF and rEF groups relative to the Ctrl values at 1.9 µm (**A**) and 2.3 µm (**B**), in permeabilized cardiomyocytes. N=18–28 cells/5-6 hearts per condition. *, p<0.05 *vs* Ctrl; †, p<0.05 *vs* pEF; ANOVA followed by Tukey *post-hoc* tests.

Supplemental Figure 3

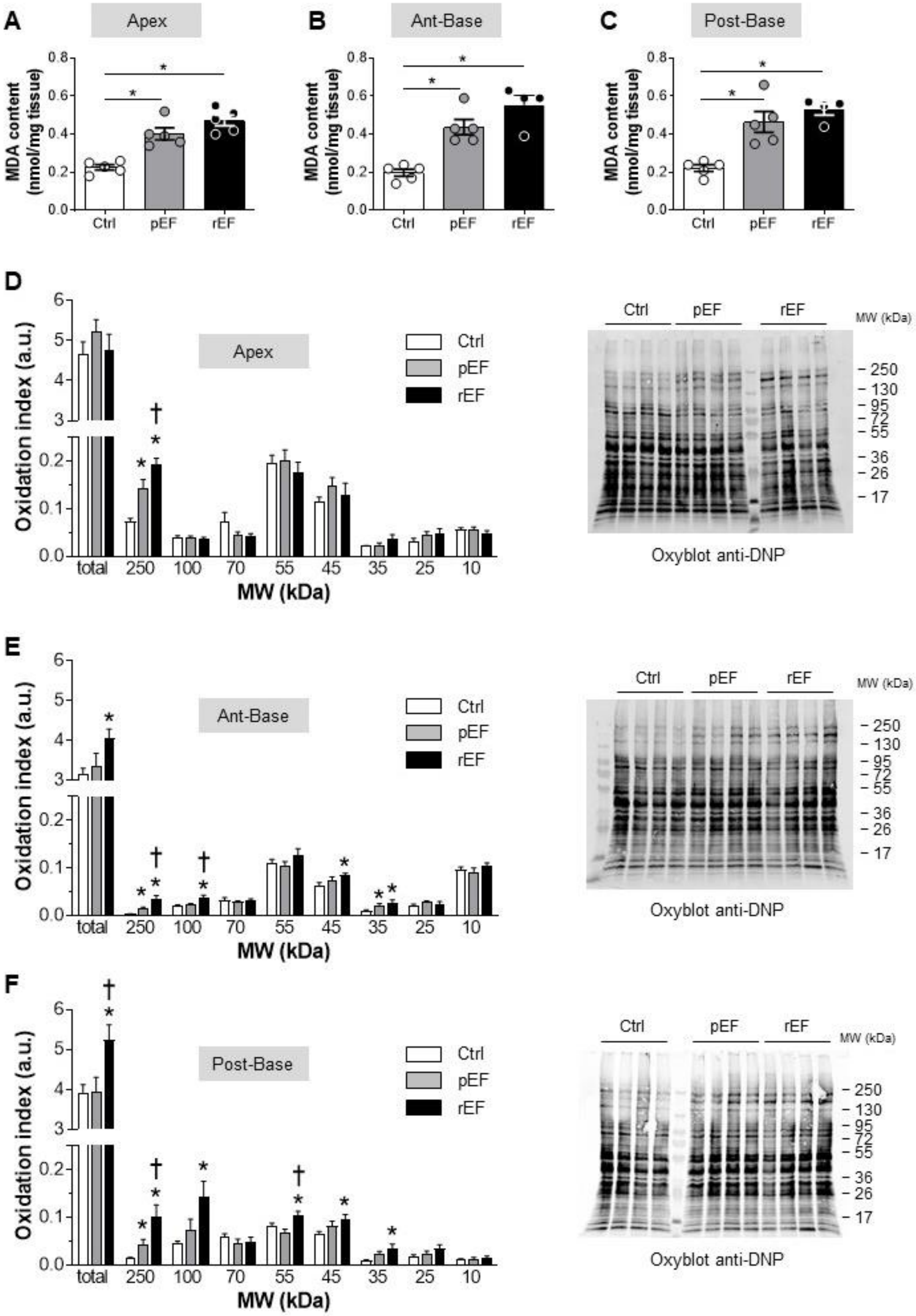

Figure S3. Regional effect of doxorubicin on oxidative stress markers at 1 month after the end of treatment. (A-C) Evaluation of lipid peroxidation based on the malondialdehyde (MDA) content in myocardial samples from LV apex **(A)**, anterior-base **(B)** and posterior-base **(C)** in Ctrl, pEF and rEF groups. (**D-F**) Carbonylation of sarcomeric proteins measured in myocardial samples from in LV apex **(D)**, anterior-base (**E**) and posterior-base **(F)** in Ctrl, pEF and rEF groups. Total proteins extracted from LV myocardium were derivatized with DNP to detect protein carbonylation. The level of carbonylated proteins was revealed by immunoblotting with anti-DNP antibodies (right panel) and was normalized to GAPDH content on the same membrane. N=4 hearts per condition. *, $p<0.05$ *vs* Ctrl; [†], $p<0.05$ *vs* pEF; ANOVA followed by Tukey *post-hoc* tests.

Full Non edited blots

Full length blots Figure 5

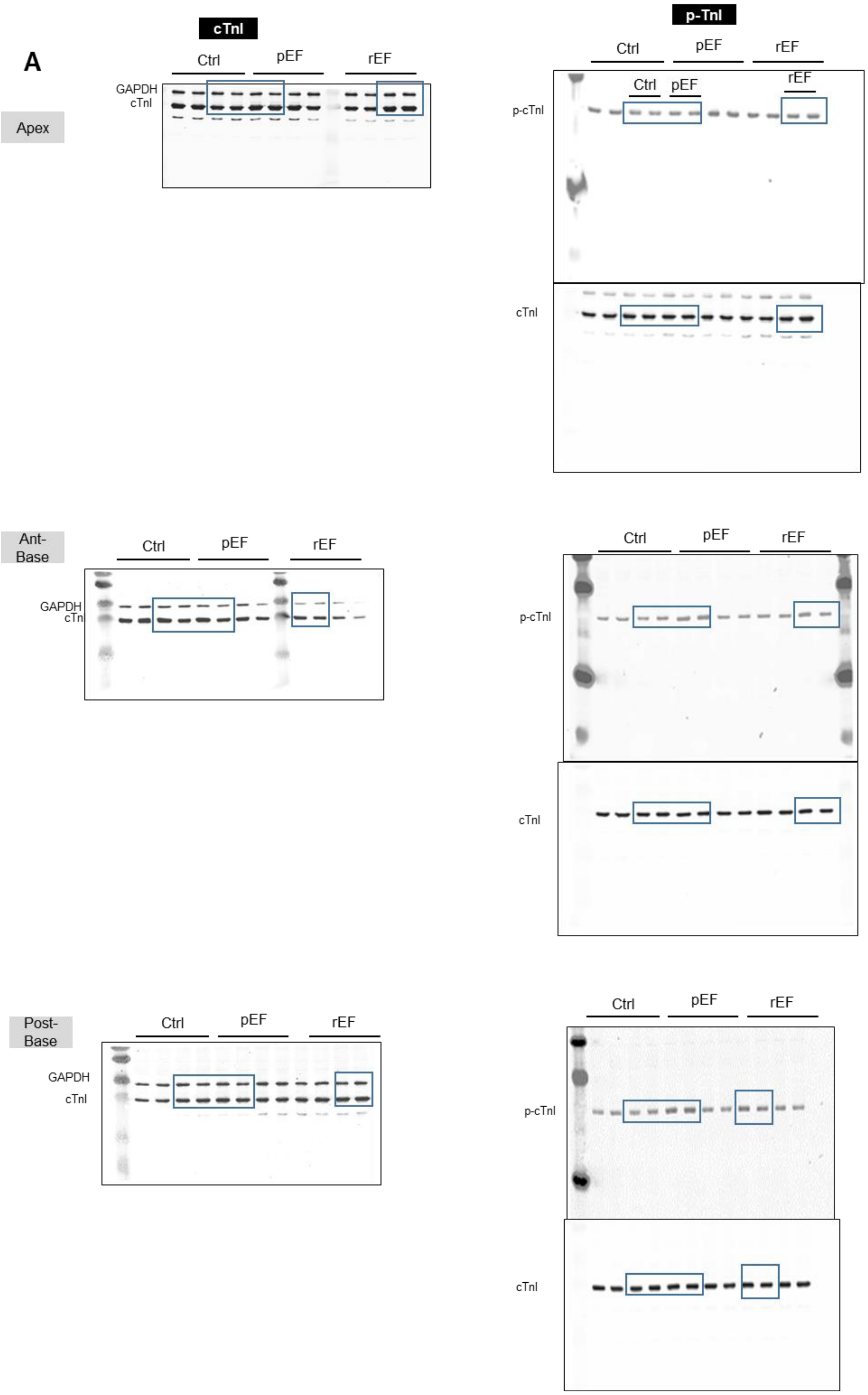

Figure 5

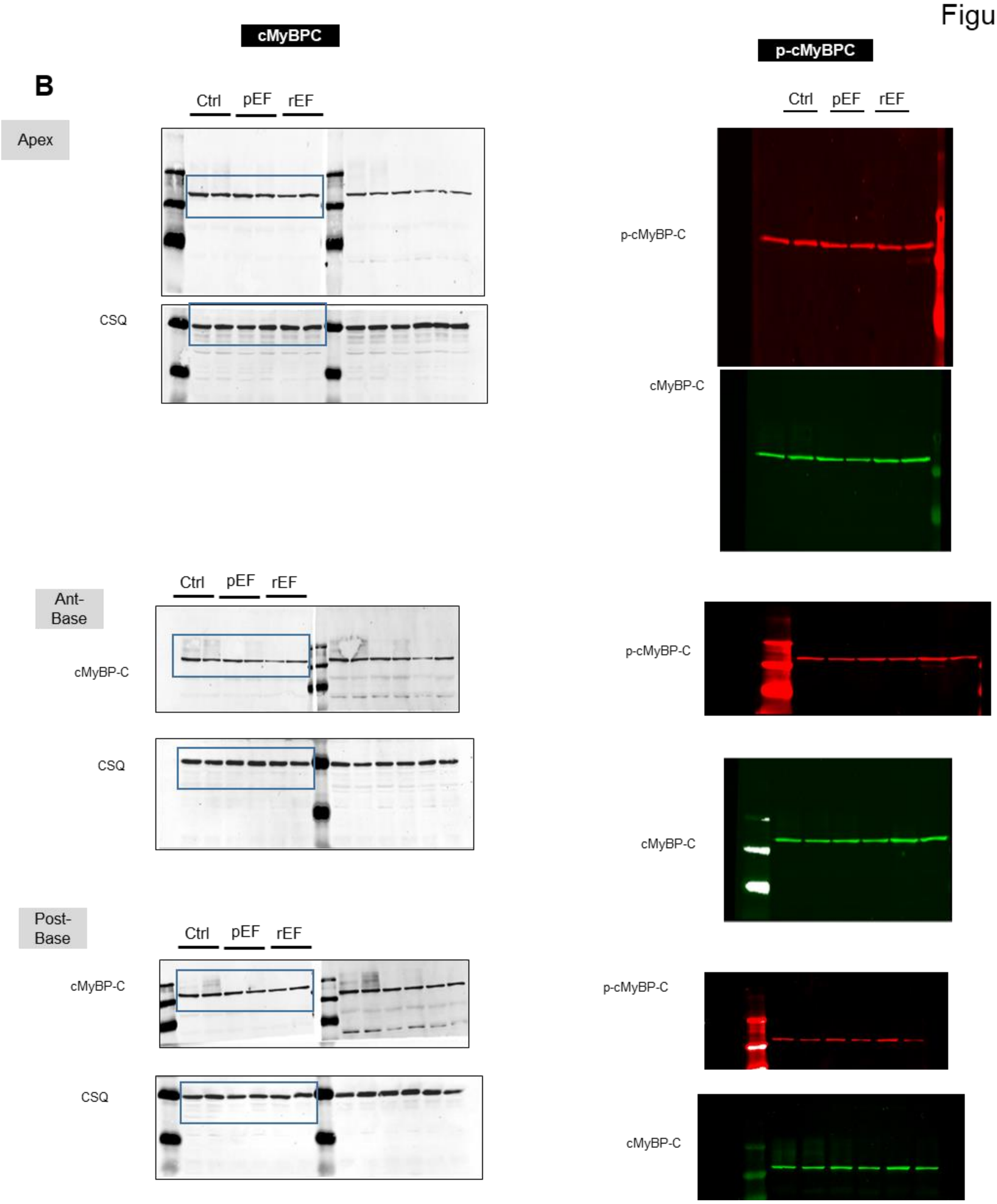
cMyBPC
p-cMyBPC
B
Apex
Ctrl
pEF
rEF
CSQ
p-cMyBP-C
cMyBP-C
Ant-Base
cMyBP-C
CSQ
p-cMyBP-C
cMyBP-C
Post-Base
cMyBP-C
CSQ
p-cMyBP-C
cMyBP-C

Figure 5

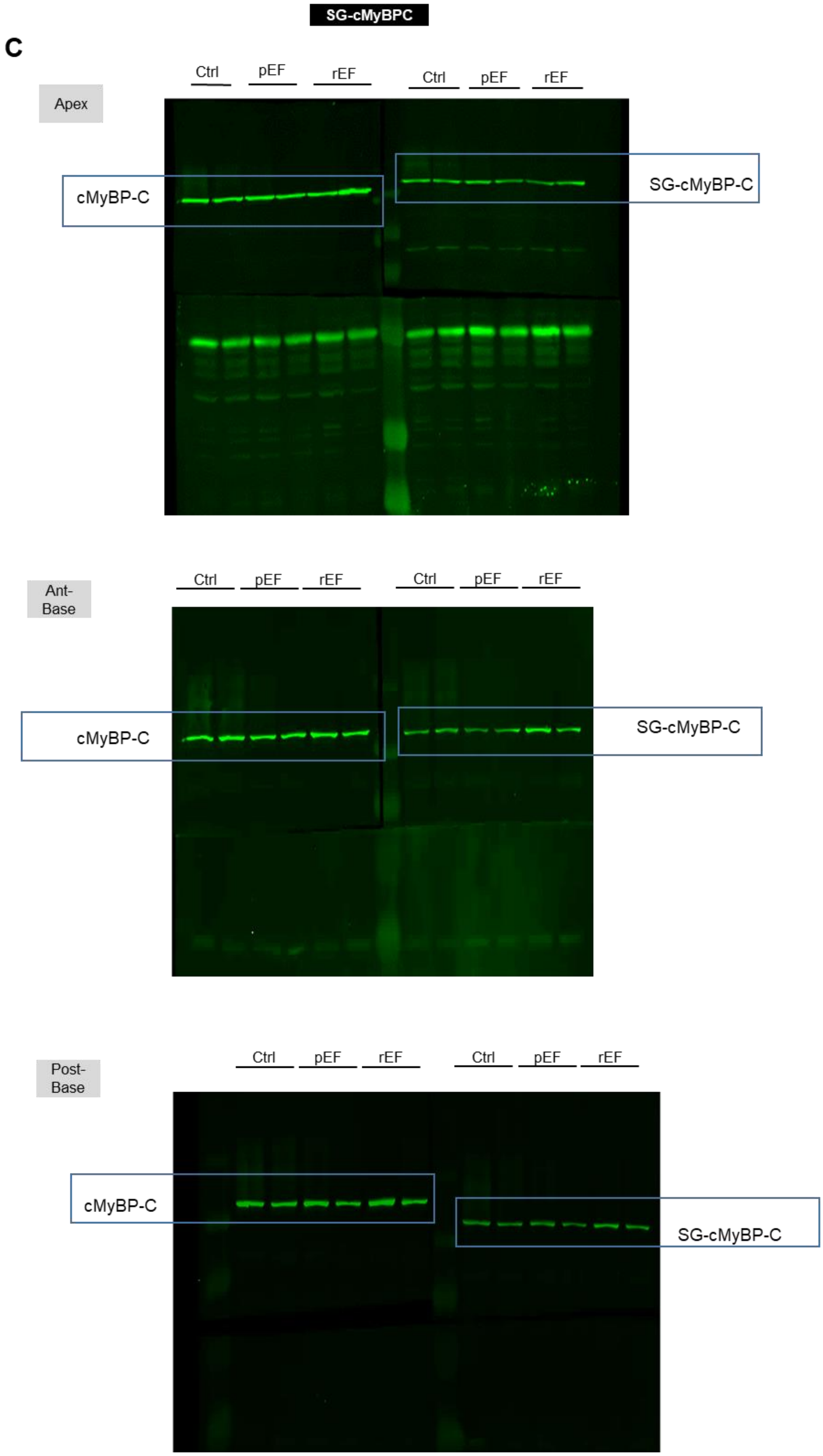
SG-cMyBPC
C
Apex
Ctrl
pEF
rEF
Ctrl
pEF
rEF
cMyBP-C
SG-cMyBP-C
Ant-
Base
Ctrl
pEF
rEF
Ctrl
pEF
rEF
cMyBP-C
SG-cMyBP-C
Post-
Base
Ctrl
pEF
rEF
Ctrl
pEF
rEF
cMyBP-C
SG-cMyBP-C

References

1. Moulin M, Piquereau J, Mateo P, et al. Sexual dimorphism of doxorubicin-mediated cardiotoxicity: potential role of energy metabolism remodeling. Circ Heart Fail. 2015; 8: 98–108.

2. Levis BE, Binkley PF, Shapiro CL. Cardiotoxic effects of anthracycline-based therapy: what is the evidence and what are the potential harms? Lancet Oncol. 2017; 18: e445–56.

3. Lang RM, Bierig M, Devereux RB, et al. Recommendations for chamber quantification. Eur J Echocardiogr J Work Group Echocardiogr Eur Soc Cardiol. 2006; 7: 79–108.

4. Andre L, Fauconnier J, Reboul C, et al. Subendocardial increase in reactive oxygen species production affects regional contractile function in ischemic heart failure. Antioxid Redox Signal. 2013; 18: 1009–20.

5. Patel BG, Wilder T, Solaro RJ. Novel control of cardiac myofilament response to calcium by S-glutathionylation at specific sites of myosin binding protein C. Front Physiol. 2013; 4: 336.

6. Chakouri N, Reboul C, Boulghobra D, et al. Stress-induced protein S-glutathionylation and phosphorylation crosstalk in cardiac sarcomeric proteins - Impact on heart function. Int J Cardiol. 2018; 258: 207–16.
